# Post-mortem diagnosis of amniotic fluid embolism

**DOI:** 10.4322/acr.2024.472

**Published:** 2024-02-02

**Authors:** Andrea Gentilomo, Stefano Tambuzzi, Guendalina Gentile, Michele Boracchi, Salvatore Andreola, Riccardo Zoia

**Affiliations:** 1 Università degli Studi di Milano, Dipartimento di Scienze Giuridiche “Cesare Beccaria”, Milano, Italia; 2 Università degli Studi di Milano, Dipartimento di Scienze Biomediche per la Salute, Laboratorio di Istopatologia e Microbiologia Forense, Istituto di Medicina Legale, Milano, Italia

**Keywords:** amniotic fluid embolism, autopsy, maternal mortality

In pregnancy, labor, delivery, or the immediate postpartum,^1^ there may be a sudden passage of amniotic fluid (containing elements of fetal origin) into the maternal bloodstream through the endocervical veins, placenta, and uterine venous sinuses.^2^ This is capable of causing a rapid and dramatic sequence of clinical events that constitute Amniotic Fluid Embolism (AFE). This event represents a rare and unpredictable complication with high mortality and morbidity^3^ and adverse perinatal outcomes, which has an incidence of maternal deaths between 5% and 24.3% in developed countries.^4^ The main risk factors are maternal age greater than 35 years, previous cesarean section, multiple pregnancies, operative delivery with forceps or suction cup, induction of labor, polyhydramnios, placental abruption, placenta previa, uterine rupture, and abdominal trauma.^5^

Although the pathogenesis and pathophysiology of AFE are not yet fully understood, it has been shown that when elements of the amniotic fluid, consisting mostly of epithelial scales detached from the skin of the fetus, downy hair, caseous varnish, mucin and/or meconium-derived bile pigments, cross the maternal-fetal barrier and enter the maternal vascular bed, triggering different adverse effects. In particular, the pathogenesis of peripheral damage derives from the obstruction of the pulmonary vessels in the first instance, subsequently aggravated by a vascular constriction on a reflex basis (vasospasm) and the appearance of diffuse microthrombosis triggered by the components of the amniotic fluid on the coagulation chain. This is typically associated with an intense anaphylactoid-like immune response to fetal or placental antigens, with a maternal systemic inflammatory syndrome^6^ that is often unstoppable. Overall, severe pulmonary hypertension and edema, marked hypoxemia, respiratory and circulatory failure, tachycardia, arterial hypotension, disseminated intravascular coagulation (DIC), multi-organ dysfunction syndrome, and, eventually, cardiac arrest may result.^7^

As far as the anaphylactoid-like reaction to fetal antigens is concerned, it is supported by the increase in serum tryptase and pulmonary mast cells in cases of AFE, as observed in post-mortem cases.^6^ This accounts for the rapid fatal course in cases characterized by even minimal AFE in which there is no mechanical obstruction of the blood flow in the maternal pulmonary vascular system.^3^

AFE still lacks a routine clinical pattern for diagnostic confirmation, and four criteria have been proposed to raise suspicion as early as possible, which include sudden cardiac arrest or respiratory and hemodynamic collapse, DIC, absence of fever, and clinical onset during labor or within 30 minutes from delivery.^8^ The diagnosis of AFE remains, however, essentially exclusionary and is strictly dependent on rapid assessment and judgment at the patient's bedside, which is not always feasible.^2^ However, a rapid initiation of adequate supportive care can increase the chance of survival of the mother and the baby.^9^

The fact remains that AFE, having sudden onset and a very often fatal outcome,^6^ remains a much-feared complication in which diagnostic certainty^2^ typically lies in the post-mortem detection of amniotic fluid elements through the mother's autopsy.^1^

**Figure 1:**
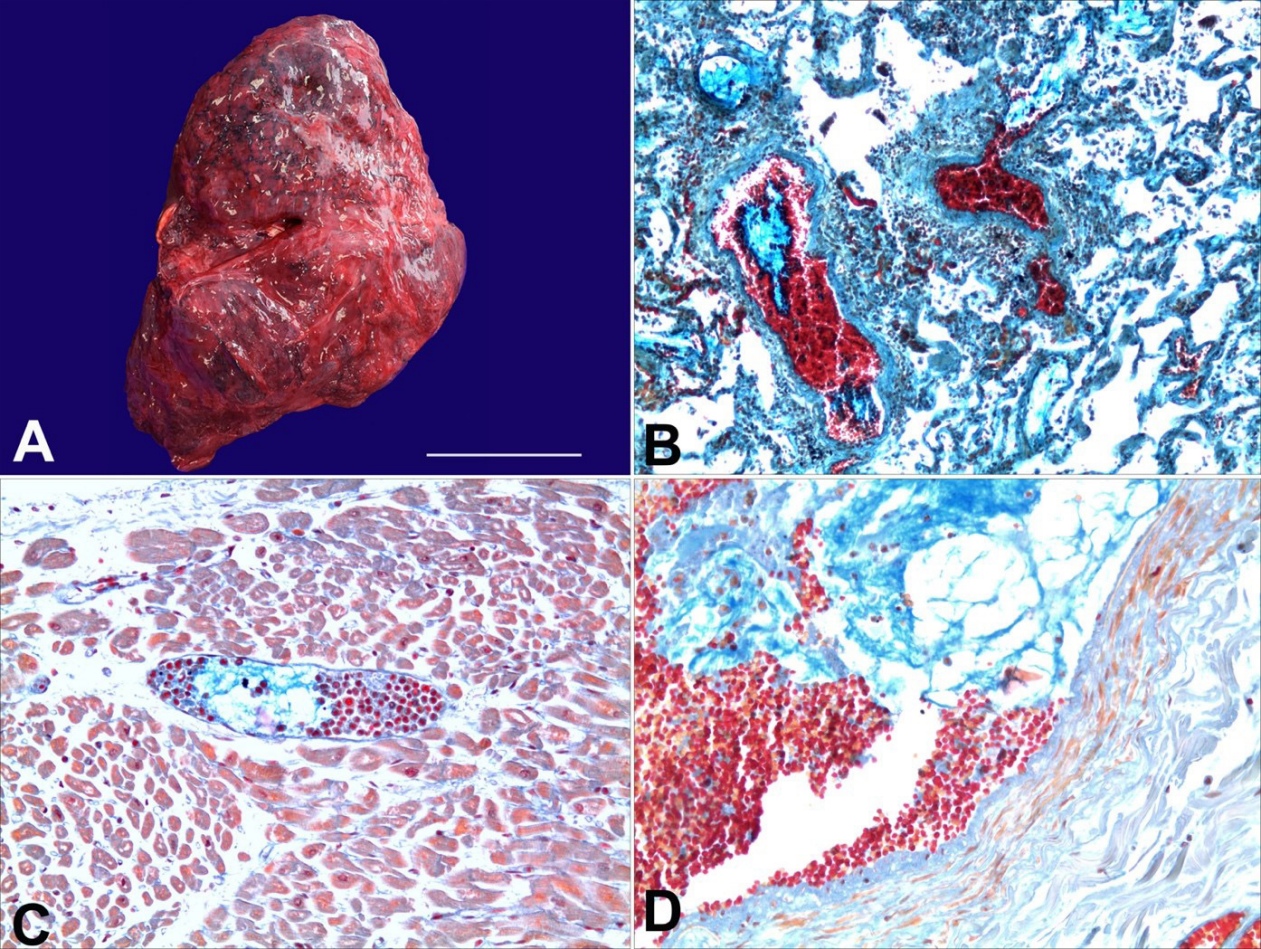
A - Gross view of the anterior surface of the right lung, which appeared reddish and with a focal area of thickened pleura (approximate size of the scale bar: 15 cm). In B, C, and D, microscopic views with differential histochemical staining of Dane and Herman,^11^ specific for the demonstration of prekeratin and keratin, visualized in orange-red, mucin visualized in turquoise, and nuclei visualized in dark-orange-brown.: in B - the presence of several mucoid emboli, poorly cellulated in pulmonary vessels, capillaries, and alveolar septa (100x); in C - the presence of mucoid material and neutrophil granulocytes in a small cardiac interfibrillar vessel (200x); in D - the presence of positive mucoid material in some small uterine intramural vessels (200x).

Figures 1A, 1B, 1C and 1D refer to a 38-year-old woman in a second pregnancy, with a previous cesarean section, admitted to the gynecology department for a scheduled second cesarean section, given the detection of a marginal posterior placenta previa. On the night before the planned surgery, the woman felt slight contractions, alerting the staff of the ward, who, upon arrival, found her responsive, with spontaneous breathing and a patent airway, without dyspnea, BP 120/80. Suddenly, however, numbness, severe arterial hypotension (BP 80/60), and tachycardia (HR 100-120) took over, requiring an emergency cesarean section performed under epidural anesthesia. After the extraction of the fetus, vital and without major complications, the woman manifested marked uterine atony without significant bleeding, marked bradycardia (38 bpm), asystole rate refractory to the administration of drugs (*atropine and* adrenaline*)* and to further resuscitation maneuvers (external cardiac massage and assisted ventilation); subsequently, the onset of ventricular fibrillation, treated with a 360J defibrillator twice, was followed by a new onset of asystole and death. Three days after her death, a judicial autopsy was performed.

At autopsy, the woman was in good condition of preservation and nutrition (weight 75.7 kg, height 154 cm), with external signs of hospitalization (acupuncture and sutured transverse abdominal surgical wound - Pfannenstiel type).

At dissection, the lungs weighed 430 g left and 500 g right (Figure 1A) [ reference range - left lung 450 g ± 146, and right lung is 494 ± 202)^10^ were congested and with diffuse pleural petechiae; when the pulmonary trunk and pulmonary vascular branches were cut, no obstruction was found. There was little blood in the pelvic cavity (55 mL). The uterus (648 g; longitudinal diameter 16 cm, transverse 14 cm, anterior-posterior 6 cm) was decidualized and showed surgical suture from the previous cesarean section between the cervical canal and the uterine body. Nothing significant emerged in the context of the other organs. The microscopic examination of the viscera fragments sampled during autopsy examination showed, using Dane and Herman staining,^11^ diagnostic findings for amniotic fluid embolism within the circulation, with emboli of mucoid material, poorly cellulated, in association with small aggregates of scales. These findings were observed in the small vessels and capillaries of the alveolar septa in the subpleural region of all five pulmonary lobes (Figure 1B), in the small intramural and subendocardial vessels, at the anterior wall of the left ventricle (Figure 1C) and in the small intramural vessels of the uterus (Figure 1D). These findings made it possible to identify the cause of the woman's death as a massive and widespread amniotic fluid embolism.

The case was brought to the attention of the scientific community because it was a sudden event characterized by embolic spread in the body, with massive involvement of the lung, heart, and uterus, which led to the death of the patient even in the absence of macroscopic obstructions to blood flow in the lungs. All this, moreover, underlines the indispensability of the post-fatal histological examination to reach the correct diagnosis of this entity, which could otherwise go unnoticed. In conclusion, amniotic embolism is unfortunately confirmed as a sudden, severe, and highly lethal obstetric complication even today, which is why both clinicians and forensic pathologists must be aware of it to implement the most appropriate diagnosis from their respective points of view.
